# Agmatine protects Müller cells from high-concentration glucose-induced cell damage via N-methyl-D-aspartic acid receptor inhibition

**DOI:** 10.3892/mmr.2015.3540

**Published:** 2015-03-24

**Authors:** NING HAN, LI YU, ZHIDU SONG, LIFU LUO, YAZHEN WU

**Affiliations:** 1 Department of Ophthalmology, The Second Hospital of Jilin University, Changchun, Jilin 130041, P.R. China; 2 Ocular Fundus Disease, The Second Hospital of Jilin University, Changchun, Jilin 130041, P.R. China

**Keywords:** agmatine, N-methyl-D-aspartic acid receptor, Müller cell, diabetic retinopathy, mitogen-activated protein kinase

## Abstract

Neural injury is associated with the development of diabetic retinopathy. Müller cells provide structural and metabolic support for retinal neurons. High glucose concentrations are known to induce Müller cell activity. Agmatine is an endogenous polyamine, which is enzymatically formed in the mammalian brain and has exhibited neuroprotective effects in a number of experimental models. The aims of the present study were to investigate whether agmatine protects Müller cells from glucose-induced damage and to explore the mechanisms underlying this process. Lactate dehydrogenase activity and tumor necrosis factor-α mRNA expression were significantly reduced in Müller cells exposed to a high glucose concentration, following agmatine treatment, compared with cells not treated with agmatine. In addition, agmatine treatment inhibited glucose-induced Müller cell apoptosis, which was associated with the regulation of Bax and Bcl-2 expression. Agmatine treatment suppressed glucose-induced phosphorylation of mitogen-activated protein kinase (MAPK) protein in Müller cells. The present study demonstrated that the protective effects of agmatine on Müller cells were inhibited by N-methyl-D-aspartic acid (NMDA). The results of the present study suggested that agmatine treatment protects Müller cells from high-concentration glucose-induced cell damage. The underlying mechanisms may relate to the anti-inflammatory and antiapoptotic effects of agmatine, as well as to the inhibition of the MAPK pathway, via NMDA receptor suppression. Agmatine may be of use in the development of novel therapeutic approaches for patients with diabetic retinopathy.

## Introduction

Diabetic retinopathy (DR) is one of the common complications associated with diabetic mellitus (DM) and it is the leading cause of blindness in people over the age of 50 (1). Recent research has demonstrated that DR is not only a microvascular disease but may be a result of neurodegenerative processes. Glucose-induced neuron and glial cell damage may occur in the absence of microvascular injury (2). Müller cells, the principal glial cells of the retina, provide neurons with adenosine triphosphate and nutrition (3), control levels of K^+^, H^+^ and neurotransmitters in extracellular space (4), and are involved in signal transmission by communicate with retinal neurons through corresponding receptors and transporters (5). Müller cells are the only cells in the retina that contain glutamine synthetase (GS). This enzyme is associated with the transformation of glutamate into glutamine via synaptic pathways (6,7). In healthy retinas, Müller cells are essential for the removal of glutamate and help to prevent the accumulation of a toxic concentration in the retina (8). In patients with DM, Müller cells are not able to transform glutamate to glutamine and, therefore, glutamate concentrations are elevated in the retinas of these individuals (9). Studies have shown that glial fibrillary acidic protein expression is upregulated and that the nucleus changes in Müller cells, during the early stages of DR (3,10–14). In addition, Müller cells secrete a number of growth factors, cytokines (15) and inflammatory regulators (16) during the development of DR, and are an important source of inflammatory factors. Therefore, the development of strategies to protect Müller cells would be beneficial in the treatment of DR.

Agmatine is an endogenous polyamine, which is formed by enzymatic decarboxylation of L-arginine in the mammalian brain (17,18). Agmatine is a neurotransmitter and neuromodulator, which is secreted from specific neuron networks (19,20). It interacts with a number of ligand-gated ion channels and binds with certain cellular receptors (21). Importantly, it is capable of inhibiting N-methyl-D-aspartic acid receptors (NMDARs) in a voltage- and concentration-dependent manner (22,23). Furthermore, agmatine inhibits nitric oxide synthase (NOS) isoforms by suppressing catalytic activity (24). Previous studies have suggested that agmatine may protect retinal ganglion cells from oxidative stress (25,26), promote glial cell survival in spinal cord injury (27) and attenuate LPS-induced microglial damage (28). However, the majority of studies have focused on the protective effects of agmatine via NMDAR inhibition in the neurons, or via NOS inhibition in the glial cells of the central nervous system (CNS). To the best of our knowledge, there have been no reports of the effects of agmatine treatment on damaged glial cells in patients with DR. NMDAR, an ionotropic glutamate receptor that mediates Ca^2+^ entry when it is activated, is present in the Müller cells of vertebrate retinas (29). Based on the data described above, agmatine is suggested to exert beneficial effects in DR damaged glial cells via NMDAR inhibition. In the present study, the protective effects of agmatine on high-concentration glucose-induced Müller cell injury were evaluated *in vitro*. The association between the protective effects of agmatine, and the expression of NMDARs and downstream signaling proteins in Müller cells was also investigated.

## Materials and methods

### Cell culture

A total of 30 male Sprague-Dawley rats, aged 4 weeks, obtained from Jilin University Laboratory Center (Jilin, China) were used for the primary Müller cells culture. The experimental protocols were approved by the ethics committee of Jilin University (Jilin, China).

Isolated retinas were washed twice using phosphate-buffered saline (PBS) and then separated using a Pasteur pipette (Xuansheng, Shanghai, China) in Dulbecco’s modified Eagle’s medium (DMEM; Gibco Life Technologies, Carlsbad, CA, USA). Cells were filtered using a cell strainer (Nanjing Union Bio-Technology, Nanjing, China), and then seeded and maintained using DMEM medium, containing 10% fetal bovine serum (Gibco Life Technologies). Cells were cultured at 37°C in humidified 5% CO_2_, and the medium was replaced every 2 days. Subsequent to 5 days of isolation, the cells were passaged every 3 days.

In order to conduct the cell survival assay, cells from the third passage were seeded into 96-well plates and divided into seven groups: (1) Healthy glucose control group (control; 25 mM glucose and 30 mM mannitol); (2) high-concentration glucose group (HG; 55 mM glucose); (3) high-concentration glucose, low-concentration agmatine treatment group (HAL; 55 mM glucose and 50 *μ*M agmatine); (4) high-concentration glucose, medium-concentration agmatine treatment group (HAM; 55 mM glucose and 100 *μ*M agmatine); (5) high-concentration glucose, high-concentration agmatine treatment group (HAH; 55 mM glucose and 200 *μ*M agma-tine); (6) high-concentration glucose, agmatine treatment and NMDA group (HAN; 55 mM glucose, 100 *μ*M agmatine and 100 *μ*M NMDA); and (7) high-concentration glucose and NMDA group (HN; 55 mM glucose and 100 *μ*M NMDA). Subsequently, cells of passage three were seeded in to 6-well or 96-well plates and divided into four groups, as the medium concentration of agmatine was considered optimal: (1) Control; (2) HG; (3) HAM; and (4) HAN.

Prior to receiving treatments, cells in each group were starved for 12 h and exposed to DMEM, containing the corresponding treatments for 48 h.

### Immunofluorescence

Müller cells were fixed on coverslips using 4% paraformaldehyde (Sinopharm Chemical Reagent Co., Ltd., Beijing, China) for 15 min, followed by washing in PBS for 5 min. Coverslips were then treated with 0.1% TritonX-100 (Sigma-Aldrich, St. Louis, MO, USA) for 30 min, at room temperature. Following a wash stage in PBS for 5 min, blocking was achieved using goat serum (Solarbio Science & Technology, Co., Ltd. Beijing, China) for 15 min at room temperature. Cells were then incubated overnight with the polyclonal NMDAR1 (1:100 dilution, catalogue no. orb99445; Biorbyt Ltd., Cambridge, UK) or GS antibodies (1:50 dilution; catalogue no. sc-9067; Santa Cruz Biotechnology, Inc., Dallas, TX, USA), at 4°C. Coverslips were washed using PBS and incubated with fluorescein isothiocyanate (FITC)-conjugated goat anti-rabbit secondary antibody (1:100; catalogue no. A0562; Beyotime Institute of Biotechnology, Haimen, China) for 1 h, at room temperature. Following a wash phase using PBS, slips were then stained with DAPI (Biosharp, Heifei, China) for 5 min. Fluorescence images were captured using a fluorescence microscope (FV1000S-SIM/IX81, Olympus Corporation, Tokyo, Japan).

### Cell survival assay

In order to conduct a cell survival assay, 10 *μ*l Cell Counting kit-8 solution (Beyotime Institute of Biotechnology) was added into each well of the plate, and plates were incubated at 37°C for 1 h. The plates were then analyzed using an enzyme-linked immunosorbent assay (ELISA) reader (ELX-800, BioTek Instruments, Inc., Winooski, VT, USA), at 450 nm.

### Lactate dehydrogenase (LDH) measurements

In order to conduct LDH activity measurements, supernatants from the Müller cells were centrifuged at 1,100 × g for 5 min. LDH activity was measured using a standard LDH kit (Nanjing Jiancheng Bioengineering Institute, Nanjing, China). Results were calculated using the following formula according to the assay kit used: LDH activity (U/L) = optical density (OD) value (sample-control)/OD value (standard-blank) × standard concentration (2 mmol/l) × 1,000.

### RNA extraction and reverse transcription-polymerase chain reaction (RT-PCR) analysis

Cells were collected and washed with PBS. Total RNA was extracted using RNA simple total RNA kit (Tiangen Biotech Co., Ltd., Beijing, China) according to the manufacturer’s instructions. cDNA was synthesized with oligonucleotide primers, using super Moloney Murine Leukemia Virus Reverse Transcriptase (BioTeke Corporation, Beijing, China). RT-PCR was performed with 1 *μ*g cDNA using the 2 × Power Taq PCR Master Mix (BioTeke Corporation) and SYBR Green (Solarbio Science & Technology, Co., Ltd.). PCR reactions were performed using a PCR system (Exicycler 96, Bioneer Corporation, Daejeon, Korea). Relative mRNA levels were normalized against β-actin and presented as 2^−ΔΔCt^. Primers used are listed in Table I.

### TNF-α expression analysis using ELISA

Following treatment for 48 h, the medium was centrifuged at 1,100 × g for 5 min and supernatants were collected in order to conduct a TNF-α assay. TNF-α expression levels were quantified according to the manufacturer’s instructions using a Rat TNF-α ELISA kit specific to rats (Multisciences, Hangzhou, China).

### Flow cytometric determination of apoptosis

Double staining using propidium iodide (PI) was performed in order to analyze annexin V-FITC binding to membrane phosphatidylserine and cellular DNA, according to the manufacturer’s instructions (Nanjing KeyGen Biotech. Co., Ltd., Nanjing, China). Following treatment for 48 h, cells were harvested and centrifuged at 88 × g for 10 min, washed twice with PBS, and resuspended in 500 *μ*l binding buffer (Nanjing KeyGen Biotech. Co., Ltd.). Annexin V-FITC (5 *μ*l) and 5 *μ*l PI were then added, and the samples were incubated for 15 min in darkness at room temperature. Samples were acquired immediately on a BD FACS Calibur flow cytometer (BD Biosciences, San Jose, CA, USA) using CellQuest software, version 3.0 (BD Biosciences). Annexin V-FITC and PI emissions were detected in the FL 1 and FL 2 channels. For each analysis, 100,000 counts were recorded. Data analysis was performed using FCS Express V3.00 (DeNovo Software, Thornhill, ON, Canada). In each plot, the lower left quadrant represented viable cells; the upper left quadrant, necrotic cells; the lower right quadrant, early apoptotic cells; and the upper right quadrant, late apoptotic cells.

### Hoechst staining

Apoptotic or necrotic cell death was characterized by staining cells using Hoechst 33342. Cells were washed twice with PBS and fixed with 5 ml fixing solution for 5 min. Subsequently, cells were stained with 10 *μ*g/ml Hoechst 33342 (Beyotime Institute of Biotechnology) for 5 min at 37°C in darkness. Cells were then washed twice with PBS and imaged using a digital camera attached to a fluorescence microscope (AE31, Motic China Group Co., Ltd., Xiamen, China).

### Western blot analysis

Cells were harvested and lysed by incubating with an NP-40 lysis buffer (Beyotime Institute of Biotechnology) containing 1% phenylmethanesulfonylfluoride on ice, for 5 min. The cells were centrifuged at 10,010 × g for 10 min at 4°C and protein concentrations were determined using a commercial bicinchoninic acid protein assay kit (Beyotime Institute of Biotechnology). Heat-induced dena-turation was conducted in a loading buffer [31% SDS (w/v), 0.67% bromophenol blue (w/v) and 33.3% glycerol (v/v)] and western blot analysis was performed, using 40 *μ*g of protein from each cell lysate. Samples and standards were loaded on an SDS gel and separated at 80 V for 2.5 h. Proteins were then transferred to polyvinylidene fluoride membranes (0.45 *μ*m; EMD Millipore, Billerica, MA, USA) in a transfer buffer [0.3% Tris (w/v), 1.44% glycine (w/v) and 20% methanol (v/v)] at 70 V for 1.5 h. Following electroblotting, membranes were washed with Tris-Buffered saline with Tween 20^®^ (TBST; Sinopharm Chemical Reagent Co., Ltd.) and blocked with TBST containing 5% non-fat milk at room temperature, for 1 h. Subsequently, the membranes were incubated in TBST containing 5% non-fat milk and primary antibodies over night, at 4°C. The following polyclonal primary antibodies were used: Bcl-2 (1:500; BA0412) and Bax (1:1,000; BA0315) (Boster Biological Technology, Wuhan, China); c-caspase-3 (cleaved-caspase-3; 1:1,000; bs-0081R; Beijing Biosynthesis Biotechnology, Beijing, China), p-ERK (AM071) and ERK (AM076) (1:1,000; Beyotime Institute of Biotechnology, Beijing, China); p-JNK (WLP006) and JNK (WLN006) (1:1,000, Wanlei Life Sciences, Shenyang, China); and p-p38 (sc-101758) and p38 (sc-7149) (1:100 and 1:200, respectively; Santa Cruz Biotechnology, Santa Cruz, CA, USA). Membranes were washed in TBST four times and incubated for 45 min at 37°C with horseradish peroxidase-linked secondary antibodies (1:5,000; Beyotime Institute of Biotechnology). Subsequently, the membranes were washed in TBST five times, incubated for 1 min in enhanced chemiluminescence solution (7 Sea Pharmtech, Shanghai, China) and then exposed to a Fuji Rx 100 X-ray film (Fuji Photo Film, Tokyo, Japan). The membranes were then incubated in stripping buffer (Beyotime Institute of Biotechnology) for 15 min at room temperature, followed by incubation and detection of the reference gene, β-actin with a polyclonal β-actin antibody (WL0001; Wanlei Life Sciences). The density of the protein bands was normalized to the β-actin signal.

### Statistical analysis

Data are expressed as the mean ± standard deviation. Multiple comparisons were analyzed using one-way analysis of variance followed by Bonferroni’s test using SPSS software, version 19 (IBM SPSS, Armonk, NY, USA). P<0.05 was considered to indicate a statistically significant difference.

## Results

### Müller cells express GS and NMDAR

**Figure 1:**
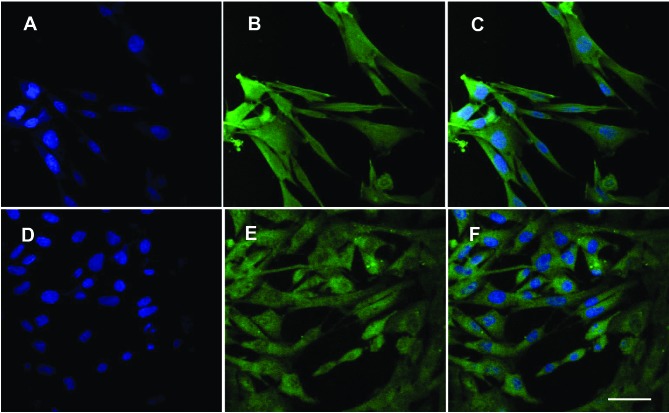
Expression of GS and NMDAR1 in Müller cells. (A) DAPI staining; (B) GS expression in Müller cells; (C) merged; (D) DAPI staining; (E) NMDAR expression in Müller cells; (F) merged. Scale bar=40 *μ*m. NMDAR, N-methyl-D-aspartic acid receptor; GS. glutamine synthetase; DAPI, 4′,6-diamidino-2-phe-nylindole; NMDAR, N-methyl-D-aspartic acid receptor.

In the retina, GS is only expressed in Müller cells. Therefore, GS was used as an indicator of the presence of Müller cells. To confirm the success of cell isolation and NMDAR expression in the cells, isolated Müller cells were immunostained using GS or NMDAR antibodies, and costained using DAPI. The nuclei were positive for DAPI (Fig. 1A, C, D and F) and the cytoplasm exhibited GS (Fig. 1B and C) or NMDAR (Fig. 1E and F) positive staining.

### Agmatine improves Müller cell survival rate

**Figure 2:**
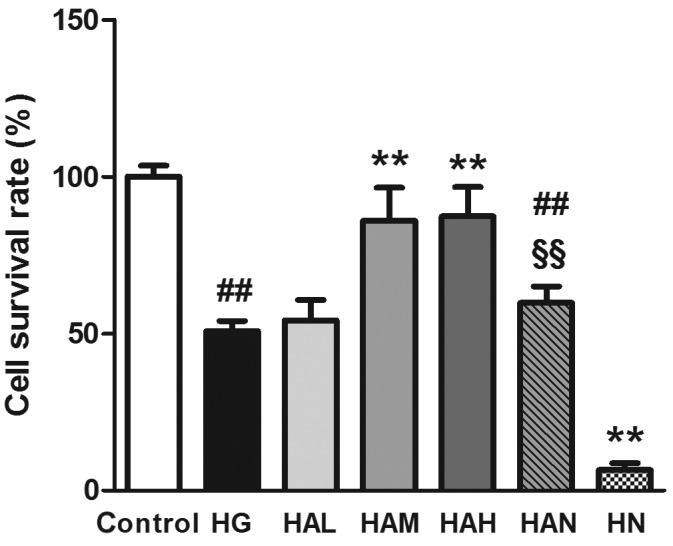
Effect of agmatine on survival rate of glucose-damaged Müller cells. Glucose treatment led to lower cell survival rates in Müller cells compared with those treated with agmatine (100 and 200 *μ*M). NMDA caused a reversal in the protective effects of agmatine treatment. Data are presented as the mean ± standard deviation (n=3). ^##^P<0.01 compared with the control group, ^**^P<0.01 compared with the HG group and ^*^P<0.01 compared with the HAM group. NMDA, N-methyl-D-aspartic acid; HAM, high-concentration glucose, medium agmatine concentration treatment; HG, high glucose.

As shown in Fig. 2, Müller cell survival was lower in cells in the HG group compared with those in the control group (50.77±3.28%, compared with 100±3.56%, P<0.01). The survival rate of Müller cells was significantly higher in cells in the HAM group compared with those in the HG group (85.98±10.46%, P<0.01). Although the mean cell survival rate value was lower, no significant difference was observed between the HAM and control groups. High-concentration agmatine treatment (HAH; 200 *μ*M) did not lead to an increase in cell survival rate compared with cells in the HAM group (100 *μ*M agmatine treatment). Following treatment with NMDA and agmatine (100 *μ*M; HAN group), the cell survival was significantly lower compared with cells in the HAM group (59.84±5.22%, P<0.01). High glucose plus NMDA treatment without agmatine (HN group) led to a significant reduction in the survival rate of Müller cells (6.52±2.20%).

### Agmatine inhibits high-concentration glucose-induced LDH activity in Müller cells

**Figure 3:**
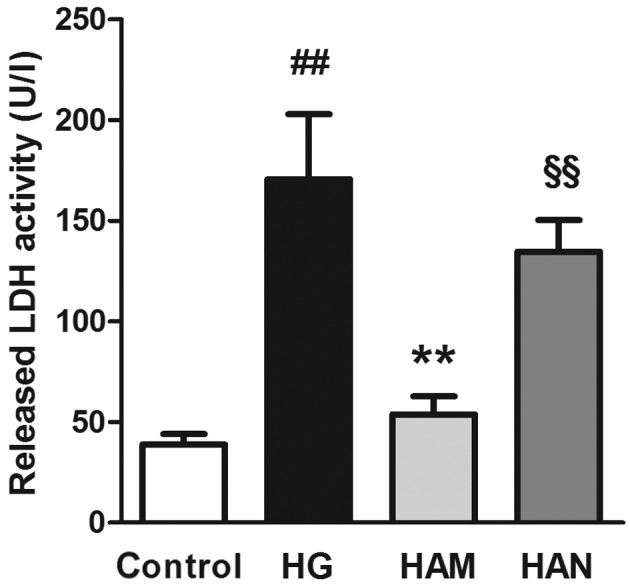
Effect of agmatine on glucose-induced LDH activity in Müller cells. Agmatine treatment led to lower LDH activity in Müller cells and NMDA treatment reversed this effect. Data are presented as the mean ± standard deviation (n=3). ^##^P<0.01 compared with the control group, ^**^P<0.01 compared with the HG group and ^#^P<0.01 compared with the HAM group. LDH, lactate dehydrogenase; NMDA, N-methyl-D-aspartic acid; HAM, high glucose, medium agmatine concentration treatment; HG, high glucose.

LDH activity was measured in order to investigate cell viability. As shown in Fig. 3, Müller cell exposure to high glucose concentrations (HG group) led to an increase in LDH activity compared with the control group (P<0.01), indicating a cellular toxic effect. LDH activity was significantly lower in Müller cells in the HAM group compared with those in the HG group (P<0.01). No significant difference was observed between the LDH activity levels of cells in the HAM group compared with those in the control group. LDH activity was significantly higher in cells in the HAN group compared with those in the HAM group.

### Agmatine inhibits high-concentration glucose-induced TNF-α release and mRNA expression

**Figure 4:**
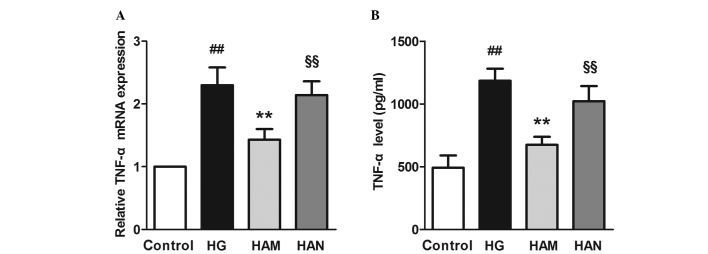
Effect of agmatine on HG-induced TNF-α inflammatory factor release. HG-induced TNF-α mRNA expression (A) and protein expression (B) in Müller cells. Agmatine treatment decreased TNF-α mRNA and protein expression levels in the culture medium, while NMDA reversed the effect of agmatine. Data are presented as the mean ± standard deviation (n=3). ^##^P<0.01 compared with the control group, ^**^P<0.01 compared with the HG group and ^#^P<0.01 compared with the HAM group. TNF-α, tumor necrosis factor-α; HG, high glucose; HAM, middle concentration of agmatine treatment; NMDA, N-methyl-D-aspartic acid.

TNF-α is a primary inflammatory factor that is released during the early stages of inflammation and is an indicator of glial cell activity. Previous studies have reported that Müller cells release TNF-α during the development of DR (30). In order to determine whether TNF-α production may be regulated by agmatine treatment, TNF-α mRNA expression and levels of TNF-α were assayed. Fig. 4 shows that TNF-α mRNA expression and TNF-α release into the medium were significantly higher in cells in the HG group compared with those in the control group (P<0.01). Agmatine treatment (HAM; 100 *μ*M agmatine) led to a significant reduction in TNF-α levels in the medium and TNF-α mRNA expression levels, compared with cells in the HG group (P<0.01). NMDA treatment (HAN group) led to significantly higher TNF-α levels in the medium and TNF-α mRNA expression levels, compared with cells in the HAM group.

### Agmatine inhibits high-concentration glucose-induced cell apoptosis in Müller cells

**Figure 5:**
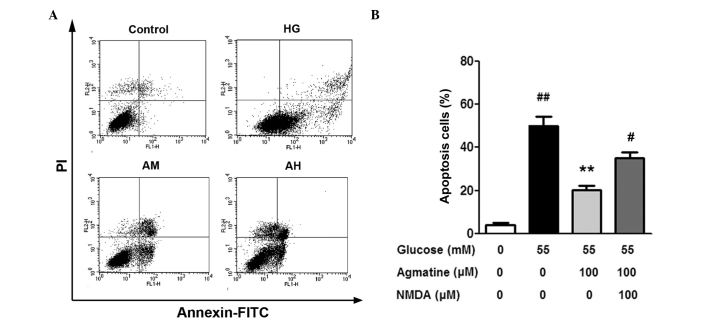
Effect of agmatine on glucose-induced apoptosis in Müller cells. (A) Flow cytometric analysis demonstrated apoptotic cells. (B) Histogram of cell apoptotic percentage. Data are presented as the mean ± standard deviation (n=3), ^##^P<0.01 compared with the control group, ^**^P<0.01 compared with the HG group and ^#^P<0.01 compared with the HAM group. HG, high glucose; HAM, high glucose, medium agmatine concentration treatment; NMDA, N-methyl-D-aspartic acid; HAH, high glucose, high agmatine concentration treatment group; PI, propidium iodide; FITC, fluorescein isothiocyanate.

A small number of cells (4.10±0.71%) were positive for Annexin V-FITC and PI staining in the control group. Following exposure to 55 mM glucose for 48 h, the percentage of apoptotic significantly increased in the HG group compared with the control group (50.07±4.30%; P<0.01; Fig. 5B). The number of apoptotic cells in the HAM group was significantly lower compared with the HG group (20.19±1.98%; P<0.01). The number of apoptotic cells was significantly higher in the HAN group compared with the HAM group (35.10±2.57%; P<0.01), suggesting that NMDA treatment suppressed the effects of agmatine on the Müller cells.

**Figure 6:**
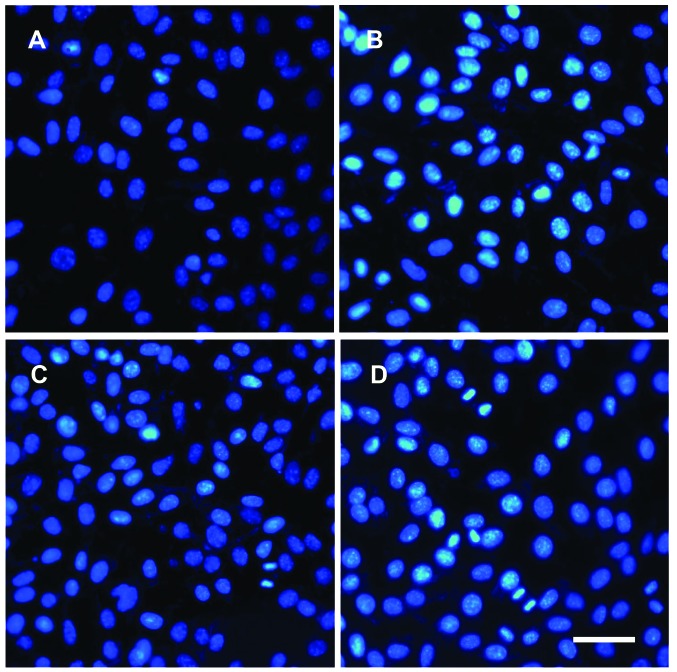
Hoechst 33342 staining in Müller cells. Agmatine treatment reduced glucose-induced cell apoptosis in Müller cells and NMDA reversed this effect. (A) Healthy control; (B) high glucose; (C) high glucose with agmatine; (D) high glucose with agmatine and NMDA. Scale bar=50 *μ*m. NMDA, N-methyl-D-aspartic acid.

Following cell apoptosis, nuclear condensation and DNA fragmentation was detected using Hoechst 33342 staining and fluorescence microscopy. As illustrated in Fig. 6, following incubation with glucose for 48 h, a number of Müller cells with condensed and fragmented nuclei were observed (Fig. 6B). Agmatine treatment (HAM group) led to a marked decrease in the number of apoptotic cells (Fig. 6C) and cells in the HAN group demonstrated nuclear condensation and DNA fragmentation following NMDA treatment. Therefore NMDA appeared to suppress the antiapoptotic effect of agmatine (Fig. 6D).

### Agmatine inhibits high-concentration glucose-induced apoptosis via regulation of apoptotic signaling protein expression

In order to further investigate the molecular mechanisms underlying the protective effects of agmatine on glucose-induced apoptosis, the expression of apoptosis-associated proteins Bax, Bcl-2 and cleaved-caspase 3 were investigated.

**Figure 7:**
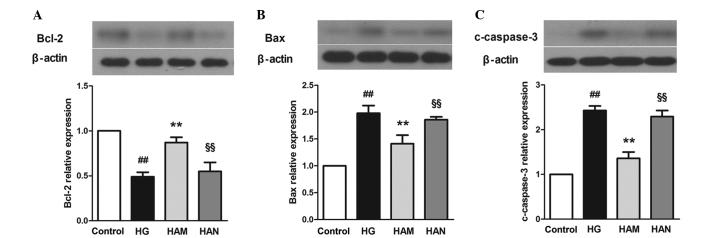
Agmatine treatment may protect cells from glucose-induced apoptosis by increasing Bcl-2 (A), and decreasing Bax (B) and c-caspase-3 (C) expression levels. NMDA reversed the effects of agmatine. Data are presented as the mean ± standard deviation (n=3). ^##^P<0.01 compared with the control group, ^**^P<0.01 compared with the HG group and ^#^P<0.01 compared with the HAM group. HG, high glucose; NMDA, N-methyl-D-aspartic acid; c-caspase-3, cleaved-caspase-3; HAM, middle concentration of agmatine treatment.

The results of the present study demonstrated that glucose treatment led to a decrease in Bcl-2 expression and an increase in Bax expression levels in Müller cells, compared with those in the control group (Fig. 7). Bcl-2 expression levels were higher and Bax expression levels were lower in the HAM group compared those the HG group. In addition, caspase-3 expression, which is an important effector of the apoptotic pathway, was higher in cells in the HG group compared with that in the control group (P<0.01). A significant decrease in caspase-3 expression was observed in cells in the HAM group compared with that in cells in the HG group (P<0.01). NMDA treatment reversed the effects of agmatine on BCl-2, Bax and caspase-3 expression in glucose-damaged Müller cells.

### Agmatine inhibits glucose-induced mitogen-activated protein kinase (MAPK) activity

**Figure 8:**
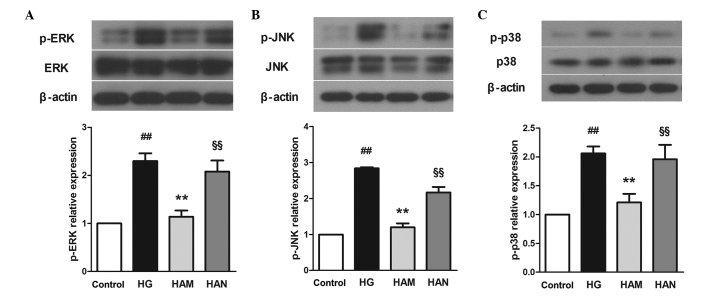
Effects of agmatine on glucose-induced MAPK signaling. Agmatine treatment may protect cells from glucose-induced cell stress by reducing ERK (A), JNK (B) and p38 (C) phosphorylation. NMDA blocked the effects of agmatine. Data are presented as the mean ± standard deviation (n=3), ^##^P<0.01 compared with the control group, ^**^P<0.01 compared with the HG group and ^#^P<0.01 compared with the HAM group. p-, phospho; c-caspase-3, cleaved-cas-pase-3; ERK, extracellular signal regulated kinase; JNK, c-Jun N-terminal kinase; p38, p38 kinase; HG, high glucose; HAM, middle concentration of agmatine treatment; NMDA, N-methyl-D-aspartic acid; MAPK, mitogen-activated protein kinase.

MAPKs are downstream proteins that are associated with the NMDAR signaling pathway. Phosphorylation levels of three MAPK-associated proteins, extracellular signal regulated kinase (ERK), c-Jun N-terminal kinase (JNK) and p38 kinase (p38), were detected in the present study. As shown in Fig. 8, ERK, JNK and p38 protein phosphorylation levels were significantly higher in cells in the HG group compared with those in the control group (P<0.01). Following agmatine treatment (100 *μ*M; HAM group), ERK, JNK and p38 phosphorylation levels were significantly decreased compared with the HG group (P<0.01). No marked changes in ERK, JNK or p38 total protein expression levels were observed (data not shown).

## Discussion

Agmatine is associated with the CNS, it interacts with certain receptors and neuronal pathways, and it demonstrates neuroprotective effects. It has been investigated for use in the treatment of CNS-associated disorders, such as spinal cord damage, ischemia, traumatic brain injury and depression (31). Agmatine has been shown to block NMDA currents in rat hippocampal neurons (23). Therefore, the effect of agmatine may be mediated via NMDA receptor inhibition (32). The protective effects of agmatine against cell damage are not restricted to the CNS; effects have also been observed in retinal ganglion cells (26,33–35). The results of the present study suggested that agmatine treatment may protect Müller cells from glucose-induced cell damage.

In the present study, glucose treatment was used to mimic DR in Müller cells. Glucose treatment induced cell death in Müller cells, and this observation was reversed following treatment with 100 and 200 *μ*M agmatine. In the present study, 100 *μ*M agmatine treatment reduced LDH activity in Müller cells in the HAM group, compared with those in the HG group. NMDA treatment reversed the protective effects of agmatine. It is possible that agmatine treatment may inhibit NMDAR, which may contribute to the protective effects of agmatine in glucose-damaged Müller cells. However, clarification of these processes requires further research.

Similar to astrocytes in the brain, Müller cells release proinflammatory cytokines in response to inflammatory responses. TNF-α is a marker of inflammation and is released during the early stages of inflammation. The results of the present study demonstrated that TNF-α expression was higher in Müller cells in the HG group compared with the control group, reflecting Müller cell inflammation. Agmatine treatment of cells in the HAM group led to a decrease in TNF-α release from Müller cells and TNF-α mRNA expression. These results were reversed following NMDA treatment. These observations are in accordance with previous studies, which showed that NMDAR treatment leads to increased TNF-α mRNA expression and secretion in cells (36,37). The results of the present study suggest that agmatine may reduce glucose-induced inflammation in Müller cells via NMDAR inhibition.

Retinal ganglion cells undergo cell apoptosis in patients with DR (38). The present study demonstrated that Müller cells undergo apoptosis in response to glucose treatment. In the present study, according to flow cytometric analysis and Hoechst staining, glucose treatment led to increased levels of Müller cell apoptosis compared with control cells. Agmatine treatment demonstrated antiapoptotic effects in glucose-damaged Müller cells. In order to investigate the mechanisms underlying the antiapoptotic effect of agmatine, signaling protein expression levels were analyzed. The Bcl-2 family consists of a group of important proteins that are involved in cell apoptosis regulation. Bcl-2 is associated with tumor development (39,40); specifically, Bcl-2 expression inhibits the morphological changes during cell apoptosis, including plasma membrane blebbing, DNA cleavage and nuclear condensation, and negatively regulates cell death (41). By contrast, Bax is a proapoptotic member of the family. A number of apoptotic signals may activate Bax expression, followed by the formation of homo-oligomers and the permea-bilization of the outer mitochondrial membrane, leading to the release of mitochondrial intermembrane contents into the cytosol (42). Bcl-2 may interact with Bax and inhibit its oligomerization, thereby inhibiting apoptosis (43). In general, the ratio of Bcl-2 to Bax proteins determines the level of cell apoptosis. Caspase-3 may be activated via extrinsic and intrinsic apoptotic pathways. Following activation by caspase-8, caspase-3 may cleave with multiple substrates within the cell, which induces cell apoptosis (44). The present study demonstrated that high-concentration glucose treatment led to a decrease in Bcl-2 expression and an increase in Bax expression, compared with control cells. Agmatine treatment promoted the upregulation of Bcl-2 and suppressed the expression of Bax, thereby contributing to a reduction in c-caspase-3 expression in cells in the HAM group. The results of the present study are consistent with a previous study that demonstrated that agmatine treatment is capable of suppressing the expression of apoptotic proteins in rat retinal ganglion cells (34). The results, therefore, suggested that Bcl-2 protein regulation may be associated with the antiapoptotic effects of agmatine.

MAPKs consist of a family of protein kinases that control a number of physiological processes and respond to various stresses signals. Three kinases, ERK, JNK and p38, are considered to be associated with stress-induced cell death. The present study demonstrated that ERK, JNK and p38 expression levels were induced in cells in the GS group, according to western blot analysis. Therefore, MAPKs may be associated with high-concentration glucose-induced Müller cell damage. In addition, MAPKs have been shown to be activated by NMDA-induced Ca^2+^ influx (45,46), and they contribute to NMDA-induced neurotoxicity in rat retinas (47). A previous study has shown that NMDAR1 expression was higher in mouse glomerular endothelial cells, following glucose treatment, compared with control cells *in vitro* (48). In the present study, increased phosphorylation of MAPKs proteins was observed in cells in the HG group compared with those in the control group. Agmatine treatment of cells in the HAM group reduced ERK, JNK and p38 phosphorylation levels, compared with cells in the HG group. ERK, JNK and p38 phosphorylation levels in cells in the HAN group were significantly lower than those in the HAM group. Therefore, glucose may induce NMDAR expression in Müller cells, which may activate MAPK protein expression. Agmatine treatment reduced the phosphorylation levels of MAPKs by inhibiting NMDAR. Therefore, the inhibition of MAPKs may partly contribute to the protective effects of agmatine in Müller cells.

In the present study, agmatine treatment was shown to increase cell survival rate, decrease LDH activity, reduce TNF-α expression, regulate apoptotic-associated Bcl-2 and Bax protein expression, and inhibit ERK, JNK and p38 protein phosphorylation, in glucose-damaged Müller cells. Therefore, agmatine may protect Müller cells from glucose-induced cell death via anti-inflammatory, antiapoptotic and MAPK signaling inactivation effects. Furthermore, protective effects of agmatine were reversed following NMDA treatment, which indicates that agmatine protection of the Müller cells may be associated with NMDAR inhibition. However, the present study did not investigate whether the effects of agmatine are associated with pathways independent of NMDAR. Furthermore, the mechanisms underlying the effects observed in the present study require further investigation. In conclusion, agmatine may be an effective for treatment in DR and is a novel therapeutic candidate for this disease.

## Figures and Tables

**Table I:** Oligonucleotide primers for RT-PCR.

Primer	Sequence (5′-3′)
TNF-α-F	TGGCGTGTTCATCCGTTCT
TNF-α-R	CCACTACTTCAGCGTCTCGT
β-actin-F	GGAGATTACTGCCCTGGCTCCTAGC
β-actin-R	GGCCGGACTCATCGTACTCCTGCTT

TNF-α, tumor necrosis factor-α; RT-PCR, reverse transcription-polymerase chain reaction; F, forward; R, reverse.
